# Concerns, Knowledge, and Practices of Dentists in Mexico Regarding Infection Control during the Coronavirus Disease Pandemic: A Cross-Sectional Study

**DOI:** 10.3390/healthcare9060731

**Published:** 2021-06-14

**Authors:** José F. Gómez-Clavel, Miguel A. Morales-Pérez, Gabriela Argumedo, Cynthia G. Trejo-Iriarte, Alejandro García-Muñoz

**Affiliations:** 1 Laboratorio de Investigación en Educación y Odontología, Facultad de Estudios Superiores Iztacala, Universidad Nacional Autónoma de México, Tlalnepantla 54090, Mexico; 2 Departamento de Cirugía Oral y Maxilofacial, Hospital Central Militar, Mexico City 11649, Mexico; miguel.amoralesperez@gmail.com; 3 Departmento de Ciencias Experimentales, CCH Azcapotzalco, Universidad Nacional Autónoma de México, Azcapotzalco 02020, Mexico; cdargumedo@yahoo.com.mx; 4 Laboratorio de Investigación Odontológica Almaraz, Facultad de Estudios Superiores Iztacala, Universidad Nacional Autónoma de México, Tlalnepantla 54090, Mexico; cynthia.belegii@gmail.com (C.G.T.-I.); alexandro_06@hotmail.com (A.G.-M.)

**Keywords:** dentist, infection control practices, knowledge, concerns, COVID-19, SARS-CoV-2, pandemic, dental practice

## Abstract

Dentists are highly exposed and vulnerable during the coronavirus disease (COVID-19) pandemic, as physical proximity to patients is necessary for effective dental examination and treatment. The objective of this study was to describe the concerns, knowledge, and infection control practices of dentists in Mexico during the COVID-19 pandemic. In this cross-sectional study conducted from 22 May 2020 to 8 July 2020, an anonymous survey was distributed to dentists, which covered information regarding dentists’ sociodemographic and professional characteristics, clinical practices during the pandemic, and perceptions regarding the application of infection prevention and control guidance for dental settings during the COVID-19 pandemic. Out of 703 respondents, 73.1% (*n* = 514) were women and 53.6% (*n* = 377) were dentists with 1–10 years of experience. Regarding the statements issued by the World Health Organization (WHO) and the Centers for Disease Control and Prevention (CDC), the responses for 11 survey items had total agreement rates >90% (high frequency); seven and nine items had moderate and low frequency of total agreement, respectively. Most dentists in this study agreed with the WHO and CDC statements and were concerned regarding the possibility of infection, despite using the protective gear.

## 1. Introduction

The spread of severe acute respiratory syndrome coronavirus 2 (SARS-CoV-2) virus has been established in most countries worldwide, including Mexico, and has led to the coronavirus disease (COVID-19) pandemic. This virus will possibly remain in our lives for a long time. It can be transmitted through direct, indirect, or close contact with the saliva, respiratory secretions, or respiratory droplets of infected persons. These droplets are usually >5–10 μm in diameter; droplets <5 μm in diameter are known as droplet nuclei or aerosols [1].

Effective prevention of oral health issues and optimal personal care remains a high priority during this COVID-19 pandemic [2]. Dentists are some of the most exposed and vulnerable healthcare professionals during this pandemic, mainly owing to the physical proximity that is necessary to effectively perform a dental examination on a patient [3]. In addition, the use of dental instruments usually generates aerosols, which can cause the air borne transmission of SARS-CoV-2 by remaining suspended in air over a long duration of time [4]. Therefore, each dental patient must be considered a possible carrier of the virus and maximum infection control measures should be applied to avoid viral transmission [3].

Dentists, as well as other health professionals, will have to continue their professional practice assuming that their everyday patients may have been infected with SARS-CoV-2, are asymptomatic, are in the incubation period and will subsequently develop symptoms, or are patients with COVID-19 infection.

In this current scenario, dentists must be able to provide adequate care by complying with the measures for infections control recommended by the World Health Organization (WHO) [2] and the Centers for Disease Control and Prevention (CDC) [5]. In Mexico, information on the number of dentists with dental practice, or verification of dentists’ adherence to the recommended measures for infections prevention, is lacking. A study carried out in a small sample reported the changes made by Mexican dentists during the pandemic [6].

However, in this study, information related to the dentists’ biosafety and economic concerns, their knowledge of protocols for infection control, and their sources of funding during the closure of their professional activity, is not addressed. Finally, we believe that the dentists’ knowledge to prevent contagions during the pandemic is important information for the development of strategies that ensure that oral health professionals in Mexico are effectively informed and implement adequate security measures. Thus, the objective of this study was to describe the concerns, knowledge, and infection control practices of dentists in Mexico during the COVID-19 pandemic.

## 2. Materials and Methods

### 2.1. Study Design and Population

This was a cross-sectional study. An anonymous survey was designed, using Google’s online survey system (Google Forms Questionnaire). The questionnaire consisted of 64 items categorized into three sections. The participating dentists were required to provide sociodemographic and professional information in Section 1. Information on the clinical practices of the participants during the COVID-19 pandemic was required in Section 2. The perception of the dentists on the application of the ‘Interim Infection Prevention and Control Guidance for Dental Settings During the COVID-19 Pandemic’ was explored in Section 3, using a Likert scale [2,4]. The questionnaire was distributed through the social networks of different dental associations between 22 May 2020 and 18 July 2020. Paramedical staff and dental students were not included in this survey.

The Ethics Committee of the Faculty of Higher Studies, Iztacala, at the National Autonomous University of Mexico granted ethical approval for this study and for the use of survey results and responses (CE/FESI/062020/1357). Informed consent to participate in the study was obtained from all participants. This study and its methods followed all relevant guidelines and regulations, in accordance with the Declaration of Helsinki.

### 2.2. Sample Size Calculation

The required sample size was 707, with an “*n*” of the study universe of 100,000, a heterogeneity of 50%, a margin of error of 2%, and a confidence level of 95%. The sample size was calculated by using the online Netquest calculator (https://www.netquest.com/es/calculadora-tamano-muestra; accessed on 20 May 2020). The response acceptance was closed (18 July 2020) when the required sample size was nearly achieved and there were no new responses for 10 days.

### 2.3. Statistical Analysis

The data were statistically analyzed using SPSS version 21.0 for Windows (IBM Corp., Armonk, NY, USA). Descriptive statistics (frequencies and percentages) were used to describe the quantitative and categorical variables. The Mann–Whitney and Kruskal–Wallis tests were used to assess differences in mean values for Likert scale items. The level of significance was set at *p* < 0.05.

## 3. Results

A total of 703 dental surgeons completed the survey; 73% of the respondents were women. Regarding the participants’ experience in dental practice, 54%, 21%, 13%, and 12.7% of the respondents had 1–10, 11–20, 21–30, and ≥31 years of experience, respectively. Regarding the type of dental practice, 6% of the respondents practiced in a government institution, 51% owned their clinic, 24% worked on contract in a private clinic, 19% underwent both institutional and private practice, and 45% of the participants were general practice dentists (Table 1).

Out of the 703 respondents, 197 stated that they worked during the national program of the Mexican Ministry of Health called “Jornada Nacional de Sana Distancia” (National Journey of Healthy Distance). The program was implemented with an aim to contain the COVID-19 pandemic in Mexico, and it was conducted between 23 March 2020 and 30 May 2020. Among dentists who reported that they closed their clinic, 197 closed their offices during March–June (134 in March, 48 in April, 7 in May, and 3 in June); 171 closed their offices following the recommendations of the Mexican Ministry of Health; and one reported that he was infected and developed COVID-19. Among the respondents who did not have a dental practice, 31% overcame their lack of income by managing their savings, 22% earned income from other activities not related to dentistry, and 18% received financial assistance from their families. The activities performed by the dentists who stayed at home were mainly hobbies (*n* = 73), exercise (*n* = 47), and spending time with family (*n* = 47); only 21 (11%) reported that they conducted classes or attended dental lectures online (Table 2).

Among the 506 dentists who reported that they worked between April and June, only 14% stated that they worked under the normal conditions, while the remaining 86% worked under different conditions. Among the 436 dentists who worked under different conditions, only 60% attended to emergencies and 26% attended to patients who were undergoing previously initiated treatments. In total, 444 dentists performed high-risk dental activities; 73% used a dental air water spray and 80% used a handpiece. The dental procedures performed by dentists who were working during the active phase of the pandemic were as follows: dental examination and prescription of non-steroidal anti-inflammatory drugs (NSAIDs) and antibiotics (69.4%), exodontia (63%), diagnosis (59%), and endodontics (46%) (Table 3).

The infection transmission control measures applied before consultation were frequent cleaning of surfaces in between consultations (97%), asking patients to rub their hands with alcohol gel (90%), and placement of disinfectant mats with 0.5% sodium hypochlorite in the clinic (70%). The personal protective equipment (PPE) that the dentists used included heat-sealed, three-layer surgical masks (47%), N95 respirators (60.3%), gloves (98%), and face shields (94%). The agents used to disinfect the surfaces of the office prior to the consultation included sodium hypochlorite solution (58%), Lysol (54%), disinfectant wipes (47%), alcohol (23.7%), and liquid detergents (17%) (Table 4).

The percentages, by which these measures increased the costs of services by 10%, 20%, and 30%, were 19%, 8%, and 3%, respectively. The main reasons some dentists did not increase the costs of dental services included solidarity with patients (41%), the fact that the budgets were previously agreed upon (15%), or because they had reserve materials (10%).

Among the dentists who worked, 61% indicated that the number of patients they saw per day was 1–3, 68% (*n* = 344) reported that they did not increase the costs of their services, and 32% increased them. Those who increased the cost of their services reported that the increase was mainly attributed to the increase in the cost of inputs (26%) and the use of more PPE (4.2%). Regarding teleconsultation, 186 dentists reported that they did not provide remote consultation. Among those who provided remote consultation, most used WhatsApp (*n* = 290), telephone calls (*n* = 214), and video conferencing (*n* = 55) (Table 5).

The perception of the dentists regarding the recommendations or statements on biosecurity in the dental clinic issued by different organizations was also measured.

Regarding general knowledge on working safely during the COVID-19 pandemic, the statement that had the highest frequency of total agreement was item 34 (“the virus that causes COVID-19 is spread primarily through respiratory drops when an infected person coughs, sneezes, or speaks”); 11 items had a percentage of total agreement > 90%, seven and nine items had a moderate and a low frequency of total agreement, respectively (Table 6).

Regarding the concerns of dentists concerning the effectiveness of preventive measures against the transmission of SARS-CoV-2, 88.5% agreed with statement 38 (“face masks do not provide complete protection against inhalation of airborne infectious agents such as SARS-CoV-2”), 86.5% agreed with statement 47 (“I am concerned that I may become infected despite using PPE”), and 88% agreed with statement 59 (“I will continue with the same measures that I have worked with during the pandemic”). These statements had the highest frequencies of agreement. In addition, the dentists were concerned regarding the economic impact of the pandemic on their clinics. Details of the economic concerns, the perception of the dentists regarding the characteristics of dental settings, and how they see the future, are summarized in Table 7.

## 4. Discussion

In this study, we aimed to describe the attitude, knowledge, and infection control practices of dentists in Mexico during the COVID-19 pandemic. Although it is still too early to be certain regarding the general trends of the clinical experiences of dentists on the transmission of COVID-19 during dental care, this study outlines their perceptions, attitudes, and concerns during the pandemic. Most of the dentists performed risky procedures, such as turning on air compressors and using handpieces. The use of the dental handpiece and triple syringe are considered biological risk factors in the transmission of COVID-19, as they favor the diffusion of aerosol particles from saliva, blood, and secretions [7,8]. In addition, since SARS-CoV-2 can persist in aerosols for up to 3 h and has a relatively long half-life of approximately 1.1–1.2 h, this aerosol production facilitates the contamination of the environment, including the dental surfaces, instruments, and appliances [9]. The most frequent activity performed in the clinic was patient screening and the prescription of antibiotics and NSAIDs, which probably led to an increase in the prescription of antibiotics during the months of the pandemic, as reported by an English team [10].

Regarding the general knowledge of dentists on working safely during the COVID-19 pandemic, most respondents indirectly showed good knowledge of concepts and attitudes as demonstrated through their degree of agreement to work according to the guidelines obtained from the scientific literature and the provisional guidelines of the CDC [5] and the WHO [2].

Our survey showed that 72% of the respondents worked during the active phase of the pandemic, a percentage that is similar to that reported for Brazilian dentists (64%) [11]. The main types of treatments performed by dentists in Mexico were related to dental pain, in line with the results of the previous work [11]. Since the start of the pandemic, teleconsultation has become an auxiliary mean of delivering dental services related to education, consultation, and triage [12]. The medium most frequently used for teleconsultation among dentists in our study was WhatsApp (58%), whereas in the study performed in Brazil, the frequency of its use was up to 70.6% [11].

Our results were similar to those reported by Kamate et al. [13], Nasser et al. [14], and Sesgin et al. [15]. These studies also reported that dentists had good knowledge and practice scores, which are important in the prevention of COVID-19. They advised dentists to follow the guidelines of the CDC [5] and the WHO [2] in their clinics, and to sensitize their staff on the best biosecurity practices to ensure that the effects of this pandemic are mitigated.

However, responses to certain items in the questionnaire reflect practices that are not in full accordance with the recommended guidelines for infection control as established in the Guidelines for Infection Control in Dental Health-Care Settings [16]. For example, regarding the guideline for heat sterilization of the dental handpiece, only 66.4% of the respondents agreed, whereas 33.7% agreed with the following statement: “To inactivate SARS-CoV-2 in the handpiece, simply immerse it in a 70% alcohol solution for 15 min.” This practice is specifically flagged as an unacceptable and unsafe sterilization method [16,17].

Another risky practice is not following the WHO recommendation when using the handpiece, which is working with four hands; only 58.7% agreed that it is important to work with an assistant, whereas 77% indicated that they can work without an assistant. In addition, regarding working with a rubber dam and using a respirator, such as N95 or FFP2, 80.4% and 84.6% agreed with the use of N95 respirators and rubber dam, respectively; both results were consistent with those reported in another survey conducted in Mexico [6]. The use of the rubber dam has traditionally been looked down on, even among endodontists [18]. However, given the risk of generating aerosols during clinical dental work, the importance of its use among general practice dentists has to be reconsidered [19,20]. Another critical point in the work of dentists is in relation to the ventilation requirements that the operating room must have. The current recommendation is that it must be well ventilated or have an air conditioning system with EPA filters [2,21].

In a survey conducted in Poland, 71.2% of the dentists who responded to the questionnaire decided to suspend clinical practice during that specific reported time [22]. In our study population, only 28% stopped working during the study period, in contrary to the results reported by Casillas et al., which stated that only 14.8% did not attend to patients [6]. Perhaps the dentists in Mexico worked during the study period because of financial need or because they are used to following infection control protocols as established in the Guidelines for Infection Control in Dental Health-Care Settings, thus, making them confident enough to work during that period [16]. In the Polish survey, the authors reported that the main factor behind the decision to suspend clinical practice was the shortage of PPE, unlike the present study in which the participants reported that the application of the general recommendations for pandemic control was the main cause of work suspension in the office. Some elements of our survey explore the concerns of dentists, which was mainly that, despite protective measures, they may become infected. The dentists in the Polish study expressed a general feeling of anxiety and uncertainty regarding the COVID-19 situation. Another study indicated that despite having a high level of knowledge and practice, dentists worldwide are in a state of anxiety and fear while working in their respective fields [23].

Knowledge of infection control practices in the dental community may enable the reaffirmation of professional training recommendations and may serve to update the programs of dental curriculums at the undergraduate and postgraduate levels as well as in continuing education courses. This would allow dentists to become aware of the possible mistakes that may occur in professional practice and would allow for the provision of a safe clinical environment for the benefit of dentists and patients.

At the time of the survey, only one dentist claimed to have been infected with SARS-CoV-2. However, as dental office care routines are activated, more dentists are likely to be affected by COVID-19, because they were infected in their dental practice or through other activities.

Regarding concerns about the efficacy of preventive measures for SARS-CoV-2, the face mask mentioned in item 38 does not provide complete protection against the inhalation of airborne infectious agents. Ideally, the transmission of SARS-CoV-2 among dentists should be monitored. We hope that the arrival of a vaccine will allow dentists to work without fear. However, this can be done only with the awareness of maximizing the use of protective barriers to avoid the transmission of infectious diseases and applying prevention protocols against the spread of COVID-19 [24]. Although the data collection was conducted within a 2 month period, it is well known that multiple revisions on the guidelines for the management of COVID-19 have been recommended from the initial stages of the pandemic by organizations, such as the CDC. These modified recommendations do not always reach the dental professionals promptly. Therefore, it is essential to monitor these changes and promote their dissemination.

The limitations of this study should be noted. As this was a cross-sectional study and the survey was conducted online, the risk of bias cannot be ruled out. In addition, no sampling technique was used to make the study representative. Therefore, the results of this survey cannot be generalized.

## 5. Conclusions

In conclusion, most of the surveyed dentists worked during the pandemic. They had a good level of knowledge regarding the transmission routes of SARS-CoV-2 and infection control measures to manage and care for patients and themselves. However, there are great concerns regarding the possibility of becoming infected and suffering from COVID-19.

## Figures and Tables

**Table 1:** Sociodemographic characteristics of the participants.

Variables		*n* = 703	%
**Sex**			
	Female	514	73
	Male	189	27
**Years of clinical experience**			
	1–10	377	54
	11–20	145	21
	21–30	92	13
	>30	89	12.7
**Type of dental practice**			
	Institutional	44	6.3
	Private, I am the owner	356	51
	Private, I am not the owner	170	24
	Institutional and private	133	19
**Specialty**			
	General practice (not specialist)	321	45
	Orthodontics	210	30
	Endodontics	48	6.8
	Pediatric dentistry	28	4
	Other types of postgraduate degree	26	3.7
	Prosthetics/rehabilitation	23	3.3
	Periodontics	22	3.1
	Oral/maxillofacial Surgery	20	2.8
	Pathology	5	0.7

**Table 2:** Activities of dentists who did not work during the active phase of the pandemic.

	*n* = 197	%
**Date of office closure or halting work**		
March	134	68
April	48	24.4
May	7	3.6
June	3	1.5
Did not respond	5	2.53
**Dental office was closed because:**		
I followed the general recommendations for the control of the pandemic	171	86.8
I found out that a colleague was infected	1	0.5
Someone close to me was infected (friend or relative)	5	2.5
No patient wanted to attend a consultation	4	2
Some third party forced me (my boss, landlord, partner, others)	2	1
I got COVID-19	1	0.5
Did not respond	13	6.6
**Source of income (*n* = 197)**		
I received income from a private clinic/office	10	5
I received income from a public clinic/clinic	11	5.6
I received income from another job not related to dentistry	44	22.3
I received financial support from a government agency	6	3
I received financial support from close family/friends	35	17.8
I managed with my savings	60	30.5
I received loans or credit (institutional, bank)	5	2.5
I received income as a teacher	21	1.5
Did not respond	5	2.5
**I dedicated my free time at home to (*n* = 197):**		
Hobbies (guitar, drawing, music, cooking, others)	73	37
Exercise/Sports	47	24
Family	47	24
Online dentistry classes, courses, or conferences	21	11
Studying or reading on my own	5	3
Watching television, series, movies	1	0.5
Did not respond	3	1.5

**Table 3:** Work patterns of dentists who worked during the active phase of the pandemic.

	*n* = 506	%
**I worked normally**	70	14
To complete unfinished treatments	131	25.9
I only attended to emergencies	305	60.2
I turned on the air compressor	444	87.7
I used dental air water spray triple syringe	367	73
I used dental handpiece	406	80.2
**Type of treatments performed during the pandemic** **(one alternative do not exclude others)**	***F***	***%***
Dental examination and prescription of NSAIDs and antibiotics	351	69.4
Exodontia	320	63.2
Diagnosis	300	59.2
Endodontics	232	45.8
Operative dentistry	208	41.1
Pathology	190	37.5
Dental prosthesis	130	25.7
Periodontics	64	12.6
Orthodontics	64	12.6

NSAIDs, non-steroidal anti-inflammatory drugs

**Table 4:** Prevention of transmission during clinical dental care (multiple selections possible).

	*n* = 506	%
**Control measures performed prior to dental care:**		
Placement of disinfectant mats with sodium hypochlorite concentrations of at least 0.5% in the clinic	355	70
Request that patients wash their hands	324	90
Request patients to rub their hands with alcohol gel	453	90
Administer a questionnaire to inquire about the places the patient visited and explore respiratory symptoms	310	61.2
Measurement of temperature	304	60
Request patients rinse hands with chlorhexidine or hydrogen peroxide	341	67.4
Frequent cleaning of surfaces in between consultations	488	96.5
**PPE used**		
Goggles	367	73
Face shield mask	476	94
Simple two-layer surgical mask	119	24
Heat-sealed three-layer surgical mask	239	47.2
N95 respirator or equivalent	305	60.3
PPE overalls	223	44
Surgical cap	437	86.4
Gloves	498	98.4
Disposable surgical boots	164	32.4
**Substances used to disinfect surfaces before care interventions**		
Lysol	275	54.3
Chlorine	291	58
Alcohol	120	23.7
Liquid detergent	88	17.4
Disinfectant wipes	238	47

PPE, personal protective equipment.

**Table 5:** Accounting in the office.

	*F*	%	*n* = 506
**Average number of patients seen**			
1–3	309	61	
4–7	132	26	
>7	47	9.3	
They did not respond	18	3.6	
**Increased the costs of their services**	162	32	
**Reasons for increasing costs**			
Increased cost of materials and equipment	132	26	
Use of more PPE	21	4.2	
Because of the risk we run during the pandemic	5	1	
**Percentage of dental services cost increase**			
10%	95	18.8	
20%	39	7.7	
30%	20	3.4	
40%	5	1	
**Reasons for not increasing costs**			
The budgets had been agreed in advance	77	15.2	
I had reserve material	51	10	
Out of solidarity with my patients and my country	209	41.3	
**Media used for remote consultation (multiple response)**			
Phone call	214	42.3	
Email	14	2.8	
WhatsApp	290	57.3	
Videoconference (Google Meet, Zoom)	55	10.9	
Social Networks (i.e., Facebook, Twitter, Blog)	40	7.9	
Messages (SMS)	35	6.9	
I do not give remote consultation	186	36.8	

PPE, personal protective equipment.

**Table 6:** General knowledge of the dentists regarding working safely during the COVID-19 pandemic (*n* = 703).

Statement	Agree with the Statement (%)	Source of Statement
The virus that causes COVID-19 is spread primarily through respiratory drops when an infected person coughs, sneezes, or speaks.	97.7	CDC-WHO
Dentists and their assistants should be considered professionals at high exposure risk, as their practice has a high potential for exposure to known or suspected sources of the virus that causes COVID-19 during specific procedures.	97.2	WHO
It is important not to use a cellphone while caring for my patients.	97	CDC
Asymptomatic individuals or those in the incubation period may also be able to transmit SARS-CoV-2.	96.7	CDC-
During dental procedures, the use of the handpiece or ultrasonic scaler and the triple syringe produce a visible aerosol containing large droplets of water particles, saliva, blood, microorganisms, and other debris.	96.7	WHO
I must consider that every patient who comes to the office is potentially a transmitter of SARS-CoV-2.	96.2	CDC
I must proactively communicate to staff and patients the need to stay home if they are ill.	95	CDC
Dentists are directly exposed to inhalation of viral particles in aerosols, where the virus can remain viable for up to 3 h.	93.6	WHO
It is important for the office to have good natural ventilation.	93.6	CDC-WHO
If a patient comes to the office and is suspected or confirmed to have COVID-19, I should defer dental treatment.	92.7	CDC
Aerosols generated by using a dental handpiece or dental air water spray travel a short distance and land on the floor and surfaces of the dental unit and on the patient.	91.5	WHO
I must record everyone’s temperature before they enter the office.	88.5	CDC
The virus survives in aerosols for hours and on some surfaces for days.	88.8	WHO
During the COVID-19 pandemic, the dental clinic or office has unique characteristics that warrant additional considerations for infection control.	85.3	CDC
It is necessary to verify that my N95 respirator has been approved by the NIOSH.	84.8	CDC
Using the rubber dam can minimize the production of aerosols contaminated with saliva and blood when I use the high-speed handpiece.	84.6	WHO
To ensure that infection control is up to high quality standards, the dental procedures must be done in an infection isolation room.	82.1	CDC
During and after the COVID-19 pandemic, the use of N95 respirators becomes necessary.	80.4	CDC-WHO
During the pandemic, I must postpone elective procedures, surgeries, and non-urgent reviews or consultations in the clinic.	78.2	CDC-WHO
Disinfection of the patient’s footwear is useful. ^1^	74.3	
It is important that the patient use a mouthwash with 1% hydrogen.	67.7	CDC
To inactivate SARS-CoV-2 in the handpiece, I have to use heat sterilization.	66.4	CDC
It is important to work with an assistant.	58.7	WHO
I can work alone without an assistant.	48.6	WHO
It is useful to use a rinse with chlorhexidine to inactivate the SARS-CoV-2 present in the mucosa and saliva.	47.4	WHO
To inactivate SARS-CoV-2 in the handpiece, simply immerse it in a 70% alcohol solution for 15 min.	33.7	CDC
It is important that the office has air conditioning.	17.8	CDC-WHO

^1^ Local guideline. COVID-19, coronavirus disease; CDC, Centers for Disease Control and Prevention; WHO, World Health Organization; NIOSH, National Institute for Occupational Safety and Health; SARS-CoV-2, severe acute respiratory syndrome coronavirus 2. There were no differences based on sex (Mann–Whitney, *p* > 0.05) or years of experience (Kruskal–Wallis, *p* > 0.05) for any of the items.

**Table 7:** Concerns regarding the COVID-19 pandemic and the effectiveness of preventive measures against its transmission.

Item	Statement	Agree with the Statement (%)	Mean	SD
38	Face mask does not provide complete protection against inhalation of airborne infectious agents, such as SARS-CoV-2.	88.5	4.3	0.9
59	I will continue with the same measures that I worked with during the pandemic.	88	3.8	1.2
47	I am concerned that I may become infected despite using PPE.	86.5	3.8	1.1
39	No data to assess the risk of transmission during dental treatment.	70	4.3	0.9
54	Surgical masks or face masks are not designed to protect the user from inhaling viral particles.	70	3.8	1
51	Standard protective measures in daily clinical practice are not effective enough to prevent the transmission of SARS-CoV-2.	70	1.8	0.9
60	It will no longer be necessary to have as many preventive measures.	4.6	4.3	0.9
	**Concerns regarding the economic impact of COVID-19 pandemic**			
62	The costs of implementing measures to ensure stricter protection barriers will increase the costs of my services.	88.8	4.1	0.9
61	I believe the economic impact on my office will be severe.	77.4	4.1	0.9

There were no differences in responses based on sex (Mann–Whitney, *p* > 0.05) or years of experience (Kruskal–Wallis, *p* > 0.05) for any of the items. COVID-19, coronavirus disease; SARS-CoV-2, severe acute respiratory syndrome coronavirus 2; SD, standard deviation; PPE, personal protective equipment.

## Data Availability

The datasets used and/or analyzed during the current study are available from the corresponding author on reasonable request.

## References

[1] Fennelly K.P. (2020). Particle sizes of infectious aerosols: Implications for infection control. Lancet Respir. Med..

[2] Considerations for the Provision of Essential Oral Health Services in the Context of COVID-19: Interim Guidance, 3 August 2020 World Health Organization. https://apps.who.int/iris/handle/10665/333625.

[3] Which Occupations Have the Highest Potential Exposure to the Coronavirus (COVID-19)? Office for National Statistics. https://www.ons.gov.uk/employmentandlabourmarket/peopleinwork/employmentandemployeetypes/articles/whichoccupationshavethehighestpotentialexposuretothecoronaviruscovid19/2020-05-11.

[4] Transmission of SARSCoV-2: Implications for Infection Prevention Precautions: Scientific Brief, 09 July 2020 World Health Organization. https://apps.who.int/iris/handle/10665/333114.

[5] Interim Infection Prevention and Control Guidance for Dental Settings during the COVID-19 Response Centers for Disease Control and Prevention. https://www.cdc.gov/coronavirus/2019-ncov/hcp/dental-settings.html.

[6] Casillas Santana M.Á., Martínez Zumarán A., Patiño Marín N., Castillo Silva B.E., Sámano Valencia C., Salas Orozco M.F. (2021). How dentists face the COVID-19 in Mexico: A nationwide cross-sectional study. Int. J. Environ. Res. Public Health.

[7] Ge Z.Y., Yang L.M., Xia J.J., Fu X.H., Zhang Y.Z. (2020). Possible aerosol transmission of COVID-19 and special precautions in dentistry. J. Zhejiang Univ. Sci. B..

[8] Peng X., Xu X., Li Y., Cheng L., Zhou X., Ren B. (2020). Transmission routes of 2019-nCoV and controls in dental practice. Int. J. Oral Sci..

[9] van Doremalen N., Bushmaker T., Morris D.H., Holbrook M.G., Gamble A., Williamson B.N., Tamin D.H., Harcourt J.L., Thornburg N.J., Gerber S.I. (2020). Aerosol and surface stability of SARS-CoV-2 as compared with SARS-CoV-1. N. Engl. J. Med..

[10] Wordley V., Shah S., Thompson W. (2020). Increased antibiotics use. Br. Dent. J..

[11] Faccini M., Ferruzzi F., Mori A.A., Santin G.C., Oliveira R.C., Oliveira R.C.G., Queiroz P.M., Salmeron S., Pini N.I.P., Sunfeld D. (2020). Dental care during COVID-19 outbreak: A web-based survey. Eur. J. Dent..

[12] Brian Z., Weintraub J.A. (2020). Oral health and COVID-19: Increasing the need for prevention and access. Prev. Chronic Dis..

[13] Kamate S.K., Sharma S., Thakar S., Srivastava D., Sengupta K., Hadi A.J., Chaudhary A., Joshi R., Dhanker K. (2020). Assessing knowledge, attitudes and practices of dental practitioners regarding the COVID-19 pandemic: A multinational study. Dent. Med. Probl..

[14] Nasser Z., Fares Y., Daoud R., Abou-Abbas L. (2020). Assessment of knowledge and practice of dentists towards coronavirus disease (COVID-19): A cross-sectional survey from Lebanon. BMC Oral Health.

[15] Sezgin G.P., ŞirinoĞlu Çapan B. (2020). Assessment of dentists’ awareness and knowledge levels on the Novel Coronavirus (COVID-19). Braz. Oral Res..

[16] Kohn W.G., Collins A.S., Cleveland J.L., Harte J.A., Eklund K.J., Malvitz D.M. (2003). Guidelines for infection control in dental health-care settings--2003. MMWR Recomm. Rep..

[17] Pinto F.M., Bruna C.Q., Camargo T.C., Marques M., Silva C.B., Sasagawa S.M., Mimica L.M.J., Graziano K.U. (2017). The practice of disinfection of high-speed handpieces with 70% w/v alcohol: An evaluation. Am. J. Infect. Control.

[18] Madarati A.A. (2016). Why dentists don’t use rubber dam during endodontics and how to promote its usage?. BMC Oral Health.

[19] Hill E.E., Rubel B.S. (2008). Do dental educators need to improve their approach to teaching rubber dam use?. J. Dent. Educ..

[20] Villani F.A., Aiuto R., Paglia L., Re D. (2020). COVID-19 and dentistry: Prevention in dental practice, a literature review. Int. J. Environ. Res. Public Health.

[21] Ashtiani R.E., Tehrani S., Revilla-León M., Zandinejad A. (2020). Reducing the risk of COVID-19 transmission in dental offices: A review. J. Prosthodont..

[22] Tysiąc-Miśta M., Dziedzic A. (2020). The attitudes and professional approaches of dental practitioners during the COVID-19 outbreak in Poland: A cross-sectional survey. Int. J. Environ. Res. Public Health.

[23] Ahmed M.A., Jouhar R., Ahmed N., Adnan S., Aftab M., Zafar M.S., Khurshid Z. (2020). Fear and practice modifications among dentists to combat novel Coronavirus Disease (COVID-19) outbreak. Int. J. Environ. Res. Public Health.

[24] Amato A., Caggiano M., Amato M., Moccia G., Capunzo M., De Caro F. (2020). Infection control in dental practice during the COVID-19 pandemic. Int. J. Environ. Res. Public Health.

